# The cost of severe haemophilia in Europe: the CHESS study

**DOI:** 10.1186/s13023-017-0660-y

**Published:** 2017-05-31

**Authors:** Jamie O’Hara, David Hughes, Charlotte Camp, Tom Burke, Liz Carroll, Daniel-Anibal Garcia Diego

**Affiliations:** 1 Faculty of Health and Social Care, University of Chester, Chester, UK; 2 HCD Economics, Daresbury, UK; 3 The Haemophilia Society, London, UK; 4 FedHemo, Madrid, Spain

**Keywords:** Cost, Burden, Haemophilia, Health economics, Health-related quality of life

## Abstract

**Background:**

Severe haemophilia is associated with major psychological and economic burden for patients, caregivers, and the wider health care system. This burden has been quantified and documented for a number of European countries in recent years. However, few studies have taken a standardised methodology across multiple countries simultaneously, and sought to amalgamate all three levels of burden for severe disease. The overall aim of the ‘Cost of Haemophilia in Europe: a Socioeconomic Survey’ (CHESS) study was to capture the annualised economic and psychosocial burden of severe haemophilia in five European countries.

A cross-section of haemophilia specialists (surveyed between January and April 2015) provided demographic and clinical information and 12-month ambulatory and secondary care activity for patients *via* an online survey. In turn, patients provided corresponding direct and indirect non-medical cost information, including work loss and out-of-pocket expenses, as well as information on quality of life and adherence. The direct and indirect costs for the patient sample were calculated and extrapolated to population level.

**Results:**

Clinical reports for a total of 1,285 patients were received. Five hundred and fifty-two patients (43% of the sample) provided information on indirect costs and health-related quality of life *via* the PSC. The total annual cost of severe haemophilia across the five countries for 2014 was estimated at EUR 1.4 billion, or just under EUR 200,000 per patient. The highest per-patient costs were in Germany (mean EUR 319,024) and the lowest were in the United Kingdom (mean EUR 129,365), with a study average of EUR 199,541. As expected, consumption of clotting factor replacement therapy represented the vast majority of costs (up to 99%). Indirect costs are driven by patient and caregiver work loss.

**Conclusions:**

The results of the CHESS study reflect previous research findings suggesting that costs of factor replacement therapy account for the vast majority of the cost burden in severe haemophilia. However, the importance of the indirect impact of haemophilia on the patient and family should not be overlooked. The CHESS study highlights the benefits of observational study methodologies in capturing a ‘snapshot’ of information for patients with rare diseases.

## Background

Haemophilia is a genetic disorder characterised by a deficiency of a clotting factor in the blood, leading to prolonged bleed events. The disease is carried on the X chromosome and primarily affects males, though female carriers of the gene may exhibit symptoms of mild haemophilia. The two forms of the condition are Haemophilia A (Factor VIII (FVIII) deficiency) and Haemophilia B (Factor IX (FIX) deficiency); Haemophilia A is approximately four times more common than Haemophilia B [1]. The global incidence of haemophilia is approximately 1 per 4,000–5,000 m﻿ale births; in a given year, approximately 400 boys are born with haemophilia in Europe [1].

Individuals with severe haemophilia – representing approximately one-third of the haemophilia population in Europe [2, 3] – have factor levels less than 1% of that expected in a healthy person. Such individuals will experience recurrent, spontaneous bleeds, often in the absence of any trauma event [4]; four-fifths of bleed events occur within the musculoskeletal system [5]. Approximately 90% of people with severe haemophilia experience chronic haemophilic joint disease, characterised by chronic inflammation and progressive joint deformity, in one or more major joints by the age of 30 [5]. As well as joint stiffness and diminished range of motion, individuals with haemophilia experience significant acute pain during bleed events and chronic pain due to arthropathy, leading to disability and impaired quality of life in more than half of cases [6].

Prior to the 1960s, when clotting factor replacement therapy (CFRT) became widely available, life expectancy of persons with haemophilia (PWH) was very low (<30 years) in comparison to that of the general male population, with haemorrhage as the most prevalent cause of death [2]. Innovations in factor production mean that PWH in nations with developed healthcare systems can now expect a near-normal quality of life and an average lifespan no more than 10 years shorter than that of an unaffected individual [3, 4]. In this current “golden era” of haemophilia care [5], management of age-related conditions has become a novel necessity for clinicians and PWH [6, 7]. Nevertheless, a continuing major risk associated with the use of CFRT is the development of inhibitors, so-called due to the development of antibodies that inhibit factor uptake. Affected individuals experience poor bleed control and subsequently higher levels of morbidity and mortality [8] and reduced HRQoL [9, 10]. The cost of treating an individual with an inhibitor can exceed EUR 1 million per year, with regimens of immune tolerance induction (ITI) therapy lasting more than 3 years in rare cases [11, 12].

The objective of the CHESS study was to estimate and extrapolate resource use, costs, and health-related quality of life (HRQOL) for adults with severe haemophilia in the five largest European economies (EU5: France, Germany, Italy, Spain and the United Kingdom (UK)). The psychological, societal, and economic burden of haemophilia has been documented for a number of European countries [13–17]; few studies have taken a standardised methodology across multiple countries simultaneously [18], and sought to amalgamate all three levels of burden for severe disease. The overall aim of the CHESS study was to capture the annualised economic and psychosocial burden of severe haemophilia in the EU5.

## Methods

### Study cohort

The study population is a sample of adult patients (18 years and over) with severe inherited haemophilia A or B (FVIII/FIX level <1 IU dL^−1^), drawn approximately in proportion to the population of individuals with haemophilia in each of the five countries participating. Individuals with acquired haemophilia or other clotting factor deficiencies (e.g. von Willebrand disease (VWD), haemophilia C) were excluded, as symptoms of vWD are generally comparable to that of mild or moderate haemophilia [19], and our sample size would preclude meaningful conclusions for other rare coagulation disorders.

### Study design

A retrospective, cross-sectional methodology was employed. Data was collected by means of two questionnaires, designed specifically for specialists and patients: the web-based case record forms (CRFs) were used to collect information on direct medical resource utilisation and clinical data based on recorded notes; and the paper patient self-completion form (PSC), given to patients following the consultation, provided complimentary socio-economic information. One hundred and thirty-nine haematologists (and haemophilia care providers (HCPs) in France) based in hospitals and clinics completed up to eight patient CRFs, with a small number providing information for up to 16 patients. In order to minimise the risk of selection bias, physicians were encouraged to recruit the next eligible patients with whom they consulted, irrespective of their reason for consultation.

All patient participants provided informed consent. The study protocol was approved by the Research Ethics Sub Committee of the Faculty of Health and Social care within the University of Chester. The approval stipulated that the study was to be carried out in correspondence with regional and relevant guidelines.

Data collected from clinicians included clinical economic and demographic information, while data collected from patients included indirect and direct non-medical resource use, HRQOL (via the EuroQol EQ-5D-3 L), work productivity impact (via the Work Productivity and Activity Impairment (WPAI) Questionnaire), and therapy adherence (via the Morisky Medication Adherence Scale 8-item (MMAS-8)).

Data was collected between December 2014 and April 2015 and captured a period of 12 months retrospectively. Estimates of healthcare utilisation and costs were then calculated for the 12-month period. The online format of the CRFs ensured that numeric responses were kept within reasonable boundaries, and that infeasible responses (such as the ability to provide two mutually exclusive responses, for instance) were kept to a minimum. Nevertheless, a small amount of post-hoc data processing was conducted based on expert clinical guidance.

#### Statistical analysis

All direct medical and non-medical costs were sourced from publically available data (Table 1). Choice of resources to be included in the CHESS study was defined by the societal and participant/family perspectives [20]. To investigate the distribution of direct costs, resource use was separated into four categories:

**Table 1:** National costs for CHESS resource units

Resource item	Baseline unit price (EUR)				
	France^c^	Germany^d^	Italy^e^	Spain^f^	UK^g^
Direct costs					
Ambulatory care					
Haematologist visit (per visit)	25.99–45.99	20.88	27.32–23.17	65.69–113.54	124.71–228.57
Nurse visit (per visit)	81.74	34.28–38.42	15.11	20.92–37.46	19.36
Other specialist visit (per visit)	14.99–45.99	7.30–228.88	18.21–27.32	16.42–160	65.91–612.03
Blood test (per test)	1.89–53.96	0.50–112.50	2.11–17.22	4.78–98.37	4.29–7.67
Other test/examination (per test)	10.79–69.00	5.50–124.60	2.19–134.27	7.49–249.21	1.69–228.24
Drug (per IU)^a^	0.72	0.85–2.08	0.62–1.23	0.39–0.90	0.44–0.84
Hospitalisation					
Target joint (per procedure)	28.81–534.40	12.02–1,719.43	33.48–1,032.91	169.75–2,156.33	1,161.93–8,397.52
Bleed event: ward stay (per day)	290.85	514.29	265	708.71	562.88
Bleed event: ICU stay (per day)	1,174.60	1,265	366	1,559.24	1,056.82
Professional caregiver (per hour)	8.30	27.43	7.39	13.66	24.56
Indirect costs^b^					
Wage (patient/caregiver) (per hour)	24.64	27.15	17	16.35	27.75
Petrol (per mile)	0.53	0.54	0.63	0.50	0.63
Scheduled ambulance (per mile)	1.36	-	-	-	-

*Note.* Ranges presented where more than one price is possible; ICU: intensive care unit; IU: International Units

^a^Drug costs sourced via Study Steering Committee liaison and correspondence with domestic drug providers

^b^Costs for OTC medications, medical devices/aids, alternative therapies, and transfer payment entitlements provided directly by the respondent

^c^Sources: Ameli, sante.gouv, ViDAL.fr, Catalogue Commun des actes médicaux

^d^Sources: Kbv.de, meinpharmaversand.de, Einheitlicher Bewertungsmaßstab, rote-liste service

^e^Sources: AIFA, agenziafarmaco.gov

^f^Sources: Oblikue e-salud, Agencia espanola de medicamentos y productos sanitarios

^g^Sources: National Schedule of Reference Costs, Electronic Medicines Compendium

1. Ambulatory activity, including: haemophilia-related visits to haematologists and other specialists, paramedical practitioners (nurse specialists, physiotherapists, diet and nutritional support, etc.), and all tests and procedures (e.g. blood tests, diagnostic imaging);
2. Haemophilia-related admissions to hospital, based on length of stay, including admission to ICU, and surgical procedure/diagnosis (where applicable):
  - bleed-related hospital admissions; and
  - procedures on ‘target’ joints – defined here as areas of chronic synovitis [21] – including arthroscopy, arthrodesis, arthroplasty, arthrocentesis, and synovectomy;
3. CFRT; and
4. Use of a professional (paid) care provider.

All factor consumption was reported by the physician. For on-demand regimens, factor consumption for the most recent 3-month period was annualised. For prophylaxis regimens, mean IU per infusion was multiplied by the weekly infusion rate, and annualised.

Direct non-medical and indirect costs were defined based on seven categories of resource use:

1. Loss of earnings by the patient (due to absenteeism and/or early retirement);
2. Loss of earnings by an informal caregiver (family or friend) (as above);
3. Transfer payments (e.g. financial aid, state allowances);
4. Over-the-counter (OTC) medications;
5. Other medical device, personal aid, or home alteration costs (e.g. walking aids, orthotics);
6. Alternative therapies (e.g. yoga/Pilates, massage, nutritionist); and
7. Transport to/from hospital attendances.

Costs associated with temporary and long-term work absence (including early retirement) were valued using the traditional human capital approach (HCA), due to its benefits in terms of transparency and comparability versus that of the newer friction cost approach (FCA) [22]. Patients who reported early retirement due to haemophilia, or who reported that they were unable to work due to haemophilia in the 3 months prior to responding, were costed at the average annual salary (stratified by country). For patients in employment, days of work lost due to bleeds across the preceding 3 months was reported and extrapolated. Whilst information regarding comorbidities was captured in the questionnaire, all direct and indirect costs were limited to those resulting directly from haemophilia-related activity.

All local currency total costs were converted to Euros using the official conversion rate as of 30^th^ May, 2015. Per-patient costs were calculated by multiplying the quantities of the resource used with the national unit price of each resource. To extrapolate the sample costs to country population level, the per-patient costs were multiplied by national prevalence weights [23]:

$$ \begin{array}{l}-{\mathrm{P}}_i\mathrm{x}\ {\mathrm{Q}}_i = \mathrm{Cos}{\mathrm{t}}_i\\ {}-\mathrm{Cos}{\mathrm{t}}_i\mathrm{x}\ \mathrm{prevalence}\ \mathrm{weights} = \mathrm{population}\ \mathrm{cost}\\ {}-\mathrm{P} = \mathrm{price},\ \mathrm{Q} = \mathrm{resource}\ \mathrm{use},\ \mathrm{and}\ \mathrm{i} = 1\hbox{--} n\left(\mathrm{wheren} = \mathrm{number}\ \mathrm{of}\ \mathrm{cost}\ \mathrm{i}\mathrm{tems}\right)\end{array} $$

#### Health-related quality of life

Patient outcomes were obtained by means of the self-administered EQ-5D-3 L, a non-disease specific instrument covering the areas of mobility, self-care, everyday activities, pain/discomfort and anxiety/depression [24]. A three-level rating scale is applied to each dimension, with a total of 245 possible health states defined. The questionnaire has been validated in many countries in Europe and is commonly used in economic evaluation and health technology assessment [25]. The values or utilities are indicated on a scale on which death has a value of 0 and perfect health a value of 1, with negative values being possible. Validated country-specific adult value sets obtained via the EuroQol website were used, with the exception of Italy, for which values were taken from literature [26].

## Results

### Patients

The demographic data from CHESS are detailed in Table 2 and the severe haemophilia prevalence data, which was used for extrapolation of sample costs, are presented in Table 3. Reports for a total of 1,285 patients were received – 996 with severe haemophilia A and 289 with severe haemophilia B representing approximately 12% and 21% of the respective populations. 552 patients (43% of the sample) provided information on indirect costs and HRQOL via the PSC. Patients were an average of 35.9 years of age (standard deviation (SD) 14.9), with the lowest average age in Germany (31.1 years), and the highest in Italy (38.9 years). The majority of patients recruited in the study (57%) were receiving CFRT via a prophylaxis regimen; 5% of patients were diagnosed with an inhibitor at the time of the study. Coinfection with HIV and/or HCV was 3% and 5%, respectively. Mean EQ-5D-3 L index score ranged from 0.59 in the UK to 0.90 in Germany (pooled average 0.76).

**Table 2:** CHESS demographic data

	Country					
	France (*N* = 272)	Germany (*N* = 194)	Italy (*N* = 280)	Spain (*N* = 218)	UK (*N* = 321)	CHESS (*N* = 1,285)
PSCs received (%)	199 (73%)	97 (50%)	123 (44%)	96 (44%)	37 (12%)	552 (43%)
Haemophilia subtype (%)						
A	202 (74%)	153 (79%)	219 (78%)	176 (81%)	246 (77%)	996 (78%)
B	70 (26%)	41 (21%)	61 (22%)	42 (19%)	75 (23%)	289 (22%)
Age (mean (SD))	36.3 (13.7)	31.1 (12.0)	38.9 (15.5)	36.0 (14.1)	36.0 (15.9)	35.9 (14.7)
Age categories (%)						
18–35	154 (57%)	135 (70%)	142 (51%)	123 (56%)	192 (60%)	746 (58%)
36–59	95 (35%)	52 (27%)	100 (36%)	78 (36%)	91 (28%)	416 (32%)
60+	23 (8%)	7 (4%)	38 (14%)	17 (8%)	38 (12%)	123 (10%)
Treatment strategy: Prophylaxis (%)	159 (58%)	117 (60%)	143 (51%)	143 (66%)	175 (55%)	737 (57%)
Inhibitor history (%)						
Never	225 (83%)	165 (85%)	233 (83%)	179 (82%)	289 (90%)	1,091 (85%)
Previously	29 (11%)	25 (13%)	38 (14%)	27 (12%)	17 (5%)	136 (11%)
Currently	18 (7%)	4 (2%)	9 (3%)	12 (6%)	15 (5%)	58 (5%)
Patients with coinfection (%)						
HIV	2 (1%)	4 (2%)	4 (1%)	9 (4%)	19 (6%)	38 (3%)
HCV	7 (3%)	5 (3%)	22 (8%)	15 (7%)	21 (7%)	70 (5%)
EQ-5D-3 L index score (mean (SD))	0.73 (0.30)	0.90 (0.12)	0.84 (0.12)	0.63 (0.37)	0.59 (0.36)	0.76 (0.28)

**Table 3:** Prevalence of severe haemophilia A and B in CHESS countries (all ages)

Country	Haemophilia A			Haemophilia B		
	Population (X) [34]	Severe (%) (Y) [43–47]	Est. severe pop. (X $\times$ Y)	Population (X) [34]	Severe (%) (Y) [43–45, 47, 48]	Est. severe pop. (X $\times$ Y)
France	5,400	34	1,836	1,201	30	360
Germany	3,422	59	2,019	644	38	245
Italy	3,779	47	1,776	750	37	278
Spain	1,679	33	554	277	33	91
UK	5,646	35	1,976	1,165	34	396
All CHESS countries			8,123			1,370

### Total economic burden of illness

**Fig. 1:**
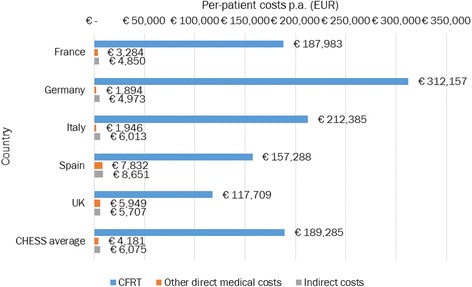
Distribution of per-patient costs (EUR). Total per-patient costs are shown for each of the five countries as well as a study average split by CFRT, other direct medical costs, and indirect costs

Total 1-year costs of severe haemophilia for the five countries was estimated to be EUR 1.55 billion (2014 values) (Table 4), with per-country costs ranging from EUR 93.8 million in Spain to EUR 700.6 million in Germany. Annual per-patient costs across the pooled sample were estimated to be EUR 199,541, ranging from EUR 129,365 in the UK to EUR 319,024 in Germany. Direct costs were estimated to be EUR 1.38 billion, representing approximately 97% of total costs and an average of EUR 193,363 per annum. Drug expenditure constitutes the vast majority (97.9%) of direct costs, a pattern that is found across all five countries (range 95.3–99.4%) (Fig. 1). Total indirect costs for the five countries were estimated to be EUR 43.3 million, averaging EUR 6,075 per patient across the pooled sample.

**Table 4:** Total economic burden of severe haemophilia in the EU5 (EUR)

Country	Total cost per country (EUR)	Total per-patient cost (EUR) (mean (SD))	Percent of total healthcare expenditures in each country
France	211,414,126	196,117	0.06%
Germany	700,257,680	319,024	0.16%
Italy	269,701,056	220,344	0.12%
Spain	94,010,111	173,771	0.05%
UK	271,278,405	129,365	0.10%
All	1,423,725,035	199,541

## Discussion

With an overall population of more than 300 million, the EU5 is home to approximately 8,123 people with severe haemophilia A and 1,370 people with severe haemophilia B. The CHESS study aimed to provide a snapshot view of the economic, societal, and psychological burden of severe haemophilia in adults within the EU5. We estimated the mean per-patient annual direct cost of severe haemophilia at EUR 173,102, EUR 313,068, EUR 210,025, EUR 132,329, and EUR 116,963 for France, Germany, Italy, Spain, and the UK, approximately 48, 79, 87, 65, and 34 times higher than the mean per-capita health expenditure in these countries, respectively [27]. Direct medical resource use was the predominant cost driver, with more than 97% of direct costs taken by CFRT, reflecting previous research suggesting that costs of CFRT account for the vast majority of the cost burden [15, 16, 28]. Annual indirect costs averaged just over EUR 6,000 per patient, with marginal variation between countries (range EUR 4,850–8,651). The total economic and societal cost of severe haemophilia in the EU5 in 2014 was estimated at EUR 1.4 billion.

Patients were recruited to the CHESS study via their haematologist or HCP, with a response rate of 43% across the pooled sample. Participation in the study by clinicians and patients was voluntary, and we therefore cannot rule out a degree of selection bias. Because the cost of illness for non-responding patients remains unknown, it is also not possible to assess the extent of the potential bias. The ratio of haemophilia A patients relative to that of haemophilia B in the CHESS cohort (approximately 1:3.5) is more narrow than current epidemiological studies, which place the ratio closer to 1:5 in severe disease [29]. The differential burden between haemophilia A and B is a topic of ongoing research; current evidence suggests that any overrepresentation by haemophilia B may generate more conservative estimates of disease burden, due to milder disease symptoms [30].

The proportion of patients receiving prophylaxis in the study cohort (57%) broadly matches that of a recent retrospective audit of haemophilia care in the EU5 [31]; prevalence of diagnosed inhibitors (4.5%) is almost identical to that of a recent study in Portugal (4.7%) [16], though the study included children (more than half of inhibitors present before the age of 9 years [32]), as well as mild and moderate haemophilia patients, who are at lower risk of inhibitor development [33].

Five percent of the CHESS cohort have recorded HCV comorbidity, with the highest rate in Italy (8%); results from the World Federation of Hemophilia Annual Global Survey [34] suggests that prevalence of HCV among haemophilia patients is upwards of one in ten, with almost half of patients in Germany co-diagnosed. Furthermore, approximately 7% of PWH in the EU5 are HIV-positive [34–36]; we find prevalence in CHESS to be less than half this amount. We suggest that a sampling bias towards non-infected patients may be present to some extent in this study. The implications of any underrepresentation for our burden estimates are uncertain: the extent to which HIV/HCV coinfection impact upon costs of haemophilia is of some dispute in current literature [37, 38], but may be more than 50% higher compared to those with haemophilia alone.

By profiling consulting patients, we draw from a cohort of patients who consult more frequently – maybe because they have difficulty with bleed control, due to joint degeneration, suboptimal therapy, or adherence. It is possible, therefore, that our sample is biased towards those older, more costly, and less adherent patients, and that our extrapolated costs may be an overestimate of the actual disease burden. However, the age distribution of the CHESS cohort is similar to that of another recent study that sourced data directly from haemophilia treatment centres and data registers across the EU5, Belgium and Sweden [31].

Our study is limited in its cost estimates by the need to use publically available reimbursement data, particularly for hospital admissions, rather than actual costs to hospital providers and patients. Any divergence between these costs may lead to an under- or over-estimate of the actual realised costs. Recall bias regarding consultations and outpatient visits may be present for clinicians, particularly for consultations with other specialists. Smaller payments by patients may also be overlooked when reporting use of devices, aids, and OTC medications. Costs of informal care provision are limited to that of the primary caregiver; informal care and household burden cost estimates were consequently underestimated for households in which additional individuals contributed to informal care.

Randomized controlled trials (RCTs) are the gold standard of evidence generation for health interventions. However, design of RCTs in rare diseases such as haemophilia is difficult due to the limited size of the patient population [39]. Further, there is often disparities between the highly structured environment of an RCT and ‘real world’ practice, particularly in the absence of formal clinical guidelines. Instead, much of the available evidence for rare diseases is based on larger-scale registries, post-authorisation surveillance studies, and observational studies such as CHESS, which have in recent years produced major insights into the treatment and management of individuals with haemophilia [40].

PWH in the five CHESS countries benefit from major investment of resources for their care and treatment. These patients are likely to obtain first access to novel long-acting and gene-based therapies in haemophilia, and it is therefore important to understand the continuing unmet need within these populations and where these new therapies will fit with current treatment approaches. The estimates of resource use and cost burden within this study are less translatable outside of Western Europe, where haemophilia often remains underdiagnosed and undertreated [41]. In countries where access to CFRT is restricted and many PWH experience major joint damage and deformity by adolescence, the burden of disease will be weighted more towards the psychosocial and employment impact resulting from severe disability and premature mortality [42].

## Conclusion

The results of the CHESS study underscore the wide variety of costs that accompany a rare disease such as haemophilia and the substantial economic burden carried by patients, caregivers, and the health care systems in these countries. Based on other existing studies of this patient group, we believe that the cost estimates from the CHESS cohort extrapolate well to the population of severe haemophilia patients in the EU5. Though the costs of CFRT account for the vast majority of the burden in this patient group, the importance of the indirect impact of haemophilia on the patient and family should not be overlooked. The CHESS study highlights the benefits of observational study methodologies in capturing a ‘snapshot’ of information for patients with rare diseases.
